# Detailed Analysis and Radiomic Prediction of First Progression Sites of First-Line Targeted Therapy for EGFR-Mutant Lung Adenocarcinoma Patients With Systemic Metastasis

**DOI:** 10.3389/fonc.2021.757892

**Published:** 2021-10-05

**Authors:** Xiaoyang Li, Runping Hou, Wen Yu, Xueru Zhu, Hongwei Li, Yidong Yang, Dong Qian, Xiaolong Fu

**Affiliations:** ^1^ Department of Radiation Oncology, The First Affiliated Hospital of The University of Science and Technology of China (USTC), Division of Life Sciences and Medicine, University of Science and Technology of China, Hefei, China; ^2^ Department of Radiation Oncology, Shanghai Chest Hospital, Shanghai Jiao Tong University, Shanghai, China; ^3^ School of Biomedical Engineering, Shanghai Jiao Tong University, Shanghai, China; ^4^ Department of Radiation Oncology, The First Affiliated Hospital of Bengbu Medical College and Tumor Hospital Affiliated to Bengbu Medical College, Bengbu, China; ^5^ Department of Engineering and Applied Physics of University of Science and Technology of China, Hefei, China

**Keywords:** stage IV lung adenocarcinoma, systemic metastasis, radiotherapy, first-line TKI, progression sites, radiomics

## Abstract

**Background:**

We aimed to analyze the first progression sites of first-line tyrosine kinase inhibitor (TKI) treatment for EGFR-mutant lung adenocarcinoma patients with systemic metastasis to recognize the potential candidates who might benefit from radiotherapy and establish a radiomic-based model to predict the first progression sites.

**Materials and Methods:**

We retrospectively collected the clinical information and pre-treatment chest CT images of patients in Shanghai Chest Hospital from 2013 to 2017. All patients were diagnosed with stage IV EGFR-mutant lung adenocarcinoma and received TKI as first-line treatment. The first progression sites and survival were analyzed. The pre-treatment chest non-contrast CT images were utilized to establish a radiomic-based model to predict the first progression sites.

**Results:**

We totally collected 233 patients with systemic metastasis, among whom, there were 84 (36.1%) and 149 (63.9%) patients developing first progression in original lesions (OP) and new lesions (NP), respectively. The PFS and OS of patients with OP were longer than those with NP (PFS 11 months *vs*. 8 months, *p* = 0.03, OS 50 months *vs*. 35 months, *p* = 0.046). For 67.9% of the patients with OF, disease progressed within five sites (oligoprogression). The radiomic-based model could predict the progression sites with an AUC value of 0.736, a specificity of 0.60, and a sensitivity of 0.750 in the independent validation set.

**Conclusion:**

Among patients with systemic metastasis, there were 36.1% of patients developing OP at first progression who had a better prognosis than those developing NP. Patients with OP may be potential candidates who might benefit from radiotherapy. Radiomics is a useful method to distinguish patients developing OP and could provide some indications for radiotherapy.

## 1 Introduction

First-line targeted therapy with tyrosine kinase inhibitor (TKI) is recommended as the standard treatment for stage IV EGFR-mutant lung adenocarcinoma. The median progression-free survival (PFS) of first-, second-, and third-generation TKI is approximately 9, 12, and 18.9 months, respectively (1–3). The acquired resistance of TKI would result in inevitable disease progression for most patients, which might be due to the dissemination of the resistant cellular clones (4). Early eradication of the potentially TKI-resistant cellular clones by local ablative modality could prolong the PFS (5). For patients with oligometastasis, the addition of radiotherapy to first-line targeted therapy has been demonstrated to be able to increase the PFS and even overall survival (OS) of EGFR-mutant patients with oligometastasis (5–7). While for patients with systemic metastasis, the role of radiotherapy has still been constrained to palliation. By far, no studies have explored whether radiotherapy could increase the PFS and even OS of first-line TKI treatment to patients with systemic metastasis. Whether radiotherapy could bring survival benefits to patients with systemic metastasis depends on the disease progression site (original lesions or new lesions) (6, 8). Radiomics is a cutting-edge technology to analyze medical imaging, which could extract discernable biological information to instruct clinical practice (9). For this purpose, we proposed to analyze the first progression sites of first-line TKI treatment for EGFR-mutant lung adenocarcinoma patients with systemic metastasis and then establish a radiomic-based model to predict the first progression sites, which might help to recognize the potential candidates who might benefit from radiotherapy.

## 2 Methods and Materials

### 2.1 Patient Enrollment

We reviewed the electric medical record of Shanghai Chest Hospital from 2013 to 2017 and collected the information of stage IV EGFR-mutant lung adenocarcinoma patients with systemic metastasis. This study was approved by the ethics committee of Shanghai Chest Hospital. The inclusion criteria were as follows: (a) EGFR mutation confirmed by ARMS or NGS; (b) stage IV with systemic metastasis confirmed by radiological examinations; (c) first-line targeted therapy with TKIs; (d) routine follow-up every 3 months with brain MRI and thoracic and abdominal CT; bone scan was undertaken every half a year; (e) accurate restaging at disease progression confirmed by brain MRI, thoracic and abdominal CT, and bone scan; and (f) available pre-treatment chest CT images with scanning thickness less than 5 mm. The exclusion criteria were as follows: (a) no EGFR mutations; (b) stage I–III, stage IV with oligometastasis; (c) other agents as first-line therapy instead of TKI; (d) no routine follow-up; (e) unknown or ambiguous metastatic patterns, PFS, OS, progression sites, and pattern; (f) local therapy, like radiotherapy or mini-invasive surgery, was added into first-line therapy; and (g) no pre-treatment chest CT images or scanning thickness over 5 mm.

### 2.2 Definition of Metastatic Pattern, First Progression Sites, Progression Pattern, and Treatment Assessment

Metastatic pattern at initial diagnosis was classified into systemic metastasis and oligometastasis. Systemic metastasis was defined as the total metastatic lesions over five sites or over three organs. Oligometastasis was defined as the total metastatic lesions less than five sites or within three organs (10). First progression sites were classified into progression in original lesions (OP) and progression in new lesions (NP) (6). OP was defined as progression within the primary or metastatic lesions, which have already existed before TKI treatment, without appearance of new metastatic lesions. NP was defined as the appearance of new metastatic lesions during TKI treatment with or without original lesion progression. Progression pattern was classified into oligoprogression and systemic progression. Oligoprogression was defined as central nervous system progression without leptomeningeal disease and amenable to whole brain radiation therapy, stereotactic radiosurgery, or surgical resection, or progression in <5 extracranial sites and amenable to SBRT, radiation therapy, or surgical resection. Systemic progression was defined as five extra-central nervous system progression sites (10, 11). TKI treatment efficacy was evaluated according to Response Evaluation Criteria in Solid Tumors (RECIST version 1.1) (12). PFS was defined as the time from the initiation of TKI treatment to disease progression or last follow-up. OS was defined as the time from the initiation of TKI treatment to death or last follow-up.

### 2.3 Establishment of Radiomic Model for the Prediction of First Progression Sites

**Figure 1:**
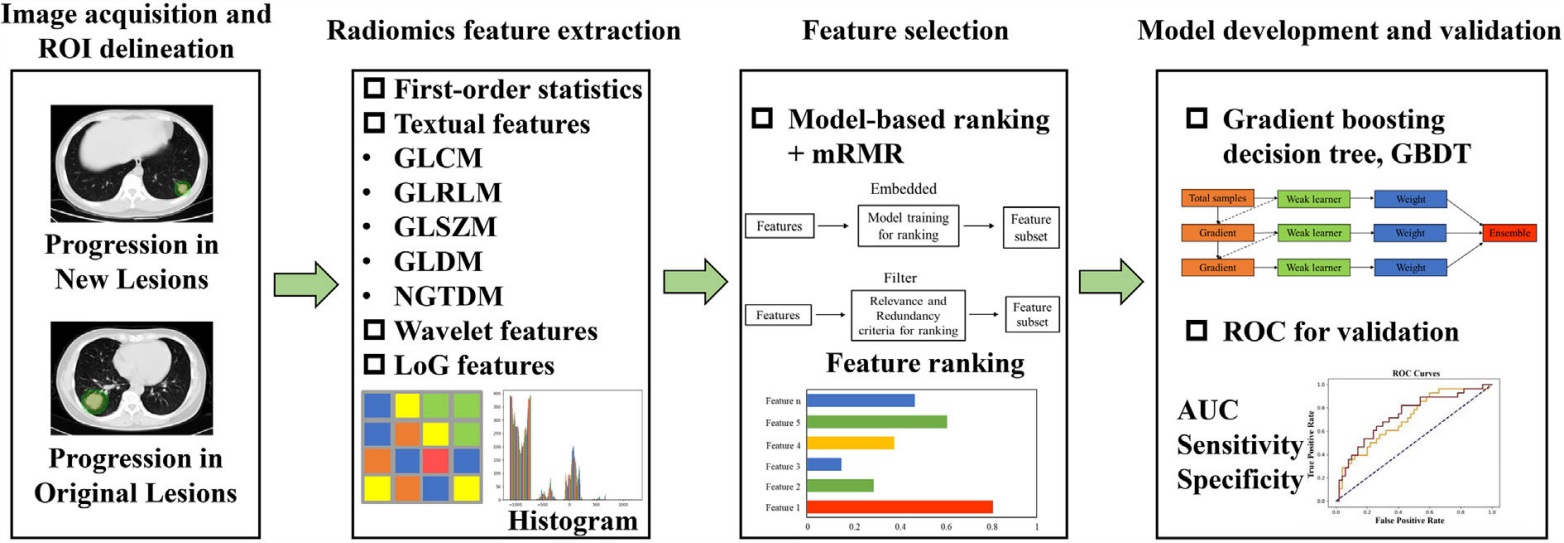
The workflow of our study. It consists of four steps: image acquisition and preprocessing, radiomic features extraction, features selection, and the classification model development and validation.

We constructed a radiomic model to predict patient’s progression sites based on the pre-treatment chest non-contrast CT scans. All the enrolled patients were randomly divided into a training set and a validation set according to the clinical characteristics. Patients with OP were regarded as positive samples with label 1 and those with NP were regarded as negative samples with label 0. The workflow of our study is shown in **Figure 1**, which consists of four steps: image acquisition and preprocessing, radiomic feature extraction, feature selection, and the classification model development and validation.

#### 2.3.1 Image Acquisition and Preprocessing

Non-contrast chest CT scans were taken with voltage from 120 to 140 kV, current 170 mA, scan layer thickness 5 mm, and spatial resolution about 1 mm using Brilliance 64 CT from PHILIPS. The contour of the primary lung lesion (region of interest, ROI) was manually delineated by an experienced radiologist (window level -400, window width 1600) on the platform Pinnalce2 for Varian^®^. CT images were linearly interpolated into 1 mm * 1 mm * 1 mm to get isotropic images.

#### 2.3.2 Radiomic Feature Extraction

High-dimensional radiomic features were extracted from the delineated three dimensional ROIs using open-source Pyradiomics package (https://pyradiomics.readthedocs.io/en/2.1.2/). Finally, a total of 1,288 radiomic features were obtained per patient, including 14 shape features, 18 first-order statistics, 73 texture features [22 gray-level co-occurrence matrix (GLCM) features; 16 gray-level run length matrix (GLRLM) features; 16 gray-level size zone matrix (GLSZM) features; 14 gray-level dependence matrix (GLDM) features; and 5 neighboring gray tone difference matrix (NGTDM) features], 728 wavelet features (8 decompositions per level, a total of 91 * 8 = 728), and 455 Laplacian of Gaussian (LoG) transformed features [sigma range:(1, 2, 3, 4, 5), a total of 91 * 5 = 455]. To eliminate the influence of different feature scales, all features were normalized to [0,1] with Min–Max scaling method before further processing.

#### 2.3.3 Feature Selection

To screen out features with good discriminability and low redundancy, two feature selection algorithms were utilized: (1) model-based ranking and (2) maximum relevance and minimum redundancy (mRMR) (13). The model-based ranking method uses each single feature to establish a prediction model of progression site. Considering that the relationship between radiomic features and the progression sites may be non-linear, we used the tree-based method (random forest) to construct the individual feature-based prediction model. Then, features were sorted and selected according to their average performance (mean AUC value) evaluated by cross-validation in the training set. The above selection process was implemented with scikit-learn package (https://scikit-learn.org/stable/). Afterwards, the mRMR method was utilized to further select features by maximizing the mutual information (MI) with the label and minimizing the average MI with all higher ranked features using Pymrmr package (https://pypi.org/project/pymrmr/). After the two steps of feature selection, radiomic features with good discrimination ability and low redundancy were finally selected for the subsequent modeling.

#### 2.3.4 Model Development and Validation

Gradient boosting decision tree (GBDT) was finally utilized to construct the prediction model using the selected radiomic features. GBDT is an ensemble learning model that obtains the final prediction results by combining multiple basic decision tree models. The model iterated continuously by gradient computing and each new learner fits into the residuals of the previous step during the training process. The hyperparameters of the GBDT model was tuned by grid search from a manually defined hyperparameter space. The optimal hyperparameter combination was determined by the average AUC value evaluated using cross-validation in the training set. When the tuning and training process completed, model performance was validated in the independent validation set to verify its generalization ability.

### 2.4 Statistical Analysis

The independent sample *t*-test and Pearson *χ* ^2^ test (or Fisher exact test) were used to compare continuous variables and categorical variables, respectively. Log-rank test was used to compare survival data. The predictive efficacy of the radiomics model was demonstrated by receiver operator characteristic curves, area under the curve (AUC) value, specificity, and sensitivity. The statistical analysis was performed on SPSS (Version 17.0) and Medcalc (Version 19.0.4). A *p*-value less than 0.05 was considered statistically significant.

## 3 Results

### 3.1 Patient Characteristics and Survival

**Figure 2:**
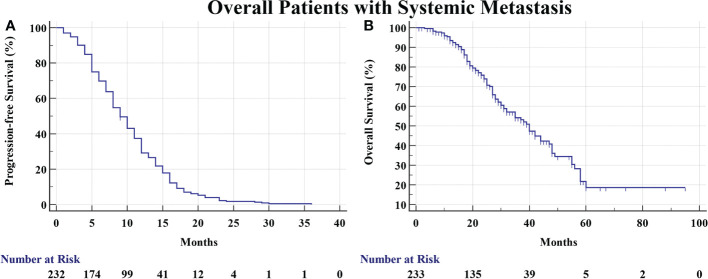
**(A)** The PFS of overall patients was 9 months. **(B)** The OS of overall patients was 40 months.

We totally screened 569 medical records and finally enrolled 233 eligible patients with systemic metastasis consecutively from 2013 to 2017 in Shanghai Chest Hospital. There were 87 (37.3%) male patients and 146 (62.7%) female patients. The median age is 61 years. There were 50 smokers (21.5%) and 183 non-smokers (78.5%). The number of patients with EGFR Exon 18, 19, 20, and 21 mutation was 1, 130, 1, and 96, respectively. There were also five patients harboring EGFR complex mutations. A total of 227 patients received first-generation TKIs (Erlotinib, Gefitinib and Icotinib) and 6 patients received afatinib. The median PFS and OS of total patients was 9 months and 40 months, respectively (**Figure 2**). There were 154 (66.1%), 127 (54.5%), 133 (57.1%), 91 (39.1%), 19 (8.2%), and 17 (7.3%) patients developing pulmonary metastasis, malignant pleural effusion, osseous metastasis, cerebral metastasis, hepatic metastasis, and adrenal metastasis at initial diagnosis, respectively (**Table 1**).

**Table 1:** Comparison of clinical characteristics of patients with progression in original lesions and progression in new lesions.

Characteristics	Overall Patients (*n* = 233)	First Progression Sites		Univariable Analysis
		Progression in Original Lesions (*n* = 84, 36.1%)	Progression in New Lesions (*n* = 149, 63.9%)	
**Gender**				
Male	87	28 (32.2%)	59 (67.8%)	Pearson *χ* ^2^ test *p* = 0.398
Female	146	56 (38.4%)	90 (61.6%)	
**Age**				
Median Age	61	59	61	*t*-test *p* = 0.603
Range	25–84	37–75	25–84	
**Smoking History**				
No smoking History	183	71 (38.8%)	112 (61.2%)	Pearson *χ* ^2^ test *p* = 0.095
Smoking History	50	13 (26%)	37 (74%)	
**PFS (months)**				
Median PFS	9	11	8	Log-rank test *p* = 0.028
Range	1–36	1–36	1–30	
**OS (months)**				
Median OS	40	50	35	Log-rank test *p* = 0.046
Range	1–95	2–95	1–88	
**Max Diameter of Primary Lung Lesion (cm)**				
Median Diameter	3.7	4.3	3.3	***t*-test** ***p* = 0.013**
Range	0.8–10.8	0.9–7.1	0.8–10.8	
>3.25 cm	145	68 (46.9%)	77 (53.1%)	**Pearson *χ* ^2^ test** ***p* = 0.0001**
≤3.25 cm	88	16 (18.2%)	72 (81.8%)	
**N Stage**				
N0	25	9 (36%)	16 (64%)	Pearson *χ* ^2^ test *p* = 0.995
N1–3	208	75 (36.1%)	133 (63.9%)	
**Mutation Site**				
Exon 19	130	52 (40%)	78 (60%)	Pearson *χ* ^2^ test *p* = 0.077
Exon 21	96	32 (33.3%)	64 (66.7%)	
Uncommon Sites	7	0	7
**Progression Pattern**				
Oligoprogression	76	57 (75%)	19 (25%)	**Pearson *χ* ^2^ test *p* = 0.0001**
Systemic Progression	157	27 (17.2%)	130 (82.8%)	
**Metastatic sites at initial diagnosis**				
Pulmonary Metastasis	154	52 (33.8%)	102 (66.2%)	Pearson *χ* ^2^ test *p* = 0.317
No Pulmonary Metastasis	79	32 (40.5%)	47 (59.5%)	
Hepatic Metastasis	19	5 (26.3%)	14 (73.3%)	Pearson *χ* ^2^ test *p* = 0.458
No Hepatic Metastasis	214	79 (36.9%)	135 (63.1%)	
Osseous Metastasis	131	41 (31.3%)	90 (68.7%)	Pearson *χ* ^2^ test *p* = 0.058
No Osseous Metastasis	102	43 (42.2%)	59 (57.8%)	
Cerebral Metastasis	91	30 (33%)	61 (67%)	Pearson *χ* ^2^ test *p* = 0.485
No Cerebral Metastasis	142	54 (38%)	88 (62%)	
Adrenal Metastasis	17	7 (41.2%)	10 (58.8%)	Pearson *χ* ^2^ test *p* = 0.794
No Adrenal Metastasis	216	77 (35.6%)	139 (64.4%)	
Plural Effusion	127	44 (34.6%)	83 (65.4%)	Pearson *χ* ^2^ test *p* = 0.682
No Plural Effusion	106	40 (37.7%)	66 (62.3%)	

The bold values are of statistical significance.

### 3.2 First Progression Site Analysis

**Figure 3:**
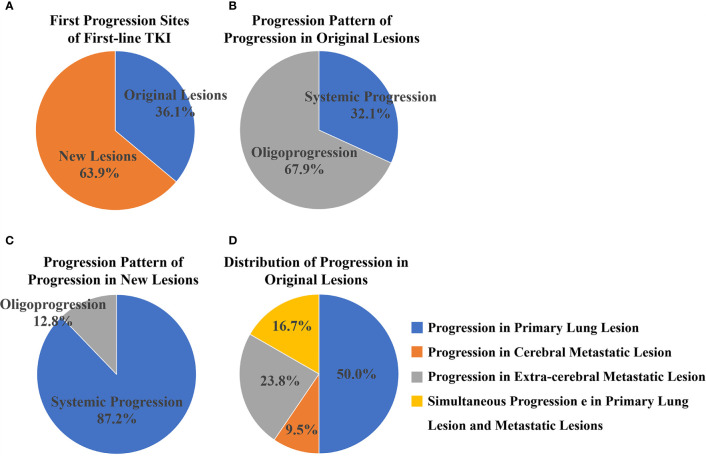
**(A)** There were 84 (36.1%) and 149 (63.9%) patients developing progression in original lesions and new lesions, respectively. **(B)** Among patients with progression in original lesions, there were 27 (32.1%) patients with systemic progression and 57 (67.9%) patients with oligoprogression. **(C)** Among patients with progression in new lesions, there were 130 (87.2%) patients with systemic progression and 19 (12.8%) patients with oligoprogression. **(D)** For patients with progression in original lesions, there were 42 (50%), 8 (9.5%), 20 (23.8%), and 14 (16.7%) patients developing progression in primary lung lesion, cerebral metastatic lesions, extra-cerebral metastatic lesions, and simultaneous primary lung lesion and metastatic lesions respectively.

**Figure 4:**
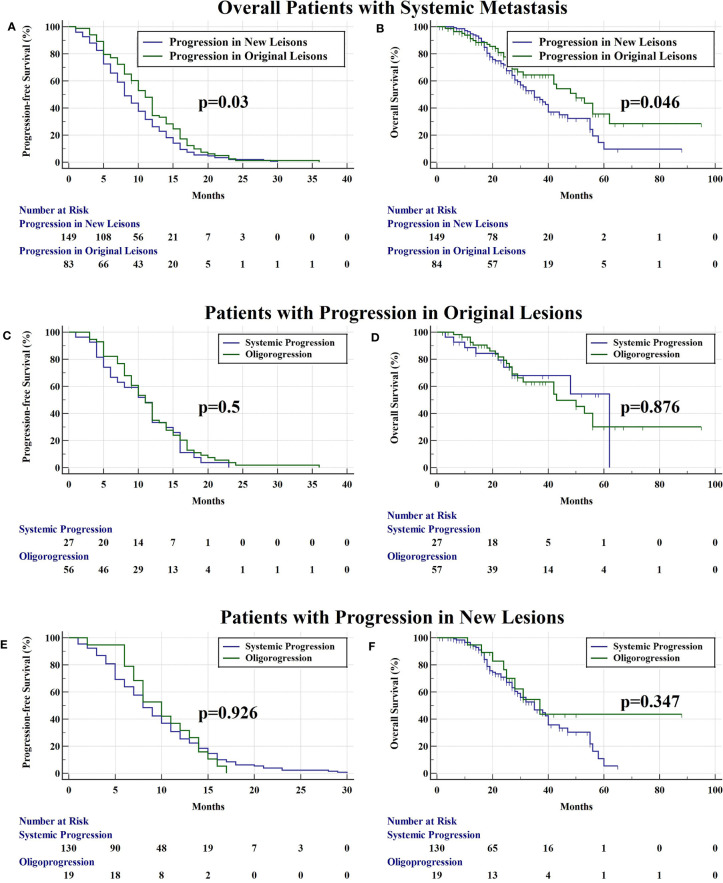
**(A)** The PFS of patients with progression in original lesions and new lesions was 11 months and 8 months (*p* = 0.03), respectively. **(B)** The OS of patients with progression in original lesions and new lesions was 50 months and 35 months (*p* = 0.046), respectively. **(C)** Among patients with progression in original lesions, there were no significant difference of PFS (11 months *vs*. 11 months, *p* = 0.500) between patients with systemic progression and oligoprogression. **(D)** Among patients with progression in original lesions, there were no significant difference of OS (62 months *vs*. 43 months, *p* = 0.876) between patients with systemic progression and oligoprogression. **(E)** For patients with progression in new lesions, there existed no significant difference of PFS (8 months *vs*. 10 months, *p* = 0.926) between patients with systemic progression and oligoprogression. **(F)** For patients with progression in new lesions, there existed no significant difference of OS (35 months *vs*. 37 months, *p* = 0.347) between patients with systemic progression and oligoprogression.

There were 84 (36.1%) and 149 (63.9%) patients developing progression in original lesions (OP) and progression in new lesions (NP), respectively (**Figure 3A**). Among patients with NP, only 15 (6.4%) patients developed new lesions with the original lesions stable. There existed no significant difference in clinical characteristics between patients with OP and NP except the max diameter of primary lung lesion and the progression pattern (**Table 1**). The max diameter of primary lung lesion of patients with OP was larger than that of NP (4.3 cm *vs*. 3.3 cm, *p* = 0.013). The cutoff max diameter of primary lung lesion to predict progression sites was 3.25 cm for the largest Youden Index. Among patients with OP, there were 27 (32.1%) patients with systemic progression and 57 (67.9%) patients with oligoprogression (**Figure 3B**). Among patients with NP, there were 130 (87.2%) patients with systemic progression and 19 (12.8%) patients with oligoprogression (**Figure 3C**). Patients with OP were more prone to develop oligoprogression. For patients with OP, there were 42 (50%), 8 (9.5%), 20 (23.8%), and 14 (16.7%) patients developing progression in primary lung lesion, cerebral metastatic lesions, extra-cerebral metastatic lesions, and simultaneous primary lung lesion and metastatic lesions, respectively (**Figure 3D**). The PFS of patients with OP and NP was 11 months and 8 months (*p* = 0.03), respectively. The OS of patients with OP and NP was 50 months and 35 months (*p* = 0.046), respectively (**Figures 4A, B**). Among patients with OP, there were no significant difference of PFS (11 months *vs*. 11 months, *p* = 0.500) and OS (62 months *vs*. 43 months, *p* = 0.876) between patients with systemic progression and oligoprogression (**Figures 4C, D**). For patients with NP, there also existed no significant difference of PFS (8 months *vs*. 10 months, *p* = 0.926) and OS (35 months *vs*. 37 months, *p* = 0.347) between patients with systemic progression and oligoprogression either (**Figures 4E, F**).

### 3.3 Radiomics Prediction of Progression Sites

**Figure 5:**
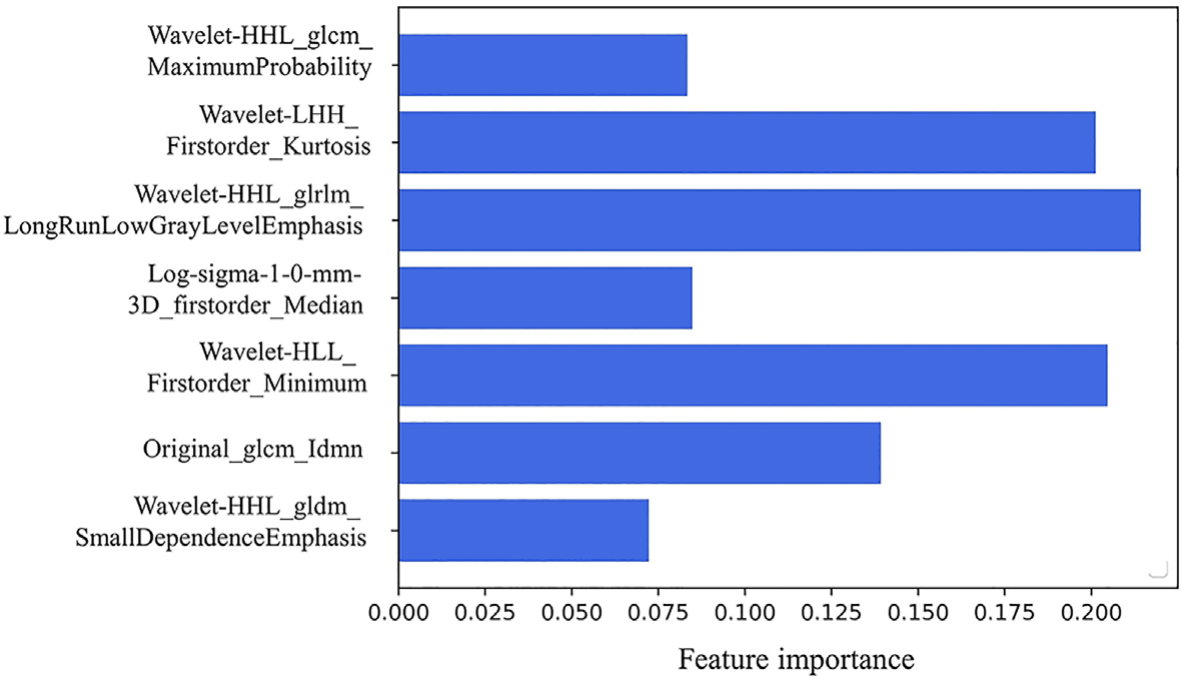
The seven selected radiomic features to establish the radiomic-based model: three wavelet-based texture features, two wavelet-based first-order features, one LoG-based first-order feature, and one original texture feature. Feature importance of the selected radiomics features. The importance coefficient in the *x*-axis was computed according to each feature’s average importance value in a single tree of the GBDT model.

**Figure 6:**
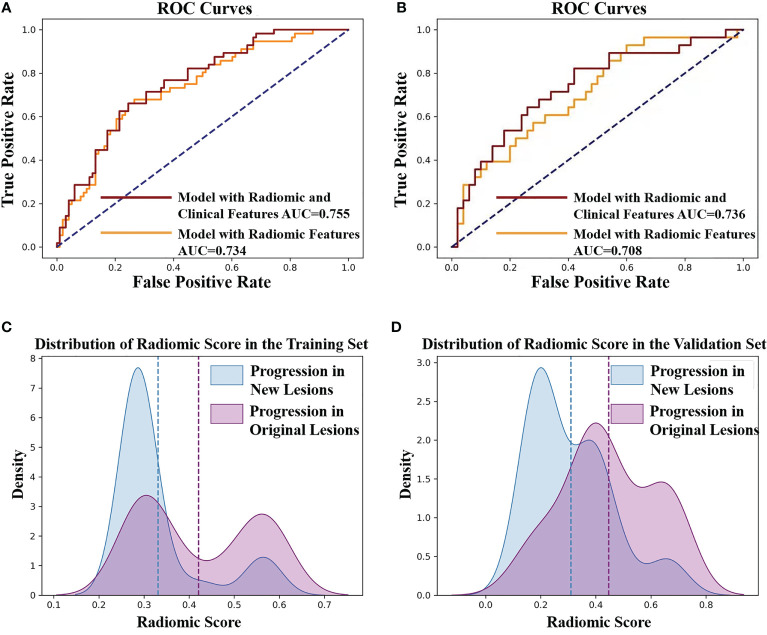
**(A)** ROC curves of the radiomic model to predict the progression sites in the training set. The AUC value of the model with radiomic features is 0.734 (yellow line). The AUC value of the model with radiomic features and clinical features is 0.755 (red line). **(B)** ROC curves of the radiomic model to predict the progression sites in the validation set. The AUC value of the model with radiomic features is 0.708 (yellow line). The AUC value of the model with radiomic features and clinical features is 0.736 (red line). **(C)** The predicted radiomic score for progression in original lesions (OP) and progression in new lesions (NP) is 0.421 ± 0.125 and 0.331 ± 0.097, respectively, in training set. **(D)** The predicted radiomic score for OP and NP is 0.447 ± 0.164 and 0.310 ± 0.146, respectively, in the validation set.

**Figure 7:**
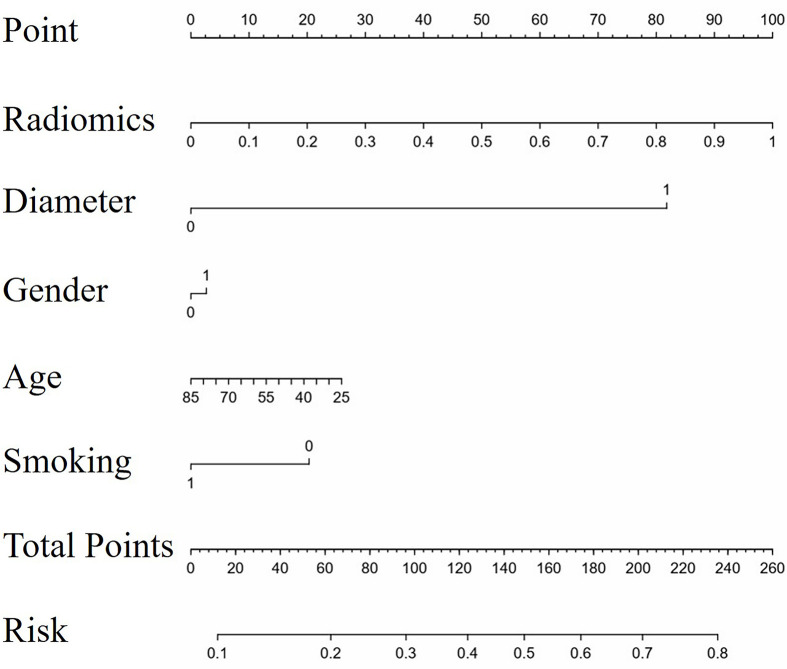
The nomogram to predict progression in original lesions. The nomogram combined radiomic score, max diameter of primary lung lesion, gender, age, and smoking history. In the axis of diameter, 0 represented the max diameter of primary lung lesion ≤ 3.25 cm and 1 represented > 3.25 cm. In the axis of gender, 0 represented male and 1 represented female. In the axis of smoking history, 0 represented non-smokers and 1 represented smokers.

There existed no significant difference of patients’ clinical characteristics between the training set and the validation set (**Table 2**). After model-based ranking followed by mRMR feature selection, seven radiomic features including three wavelet-based texture features, two wavelet-based first-order features, one LoG-based first-order feature, and one original texture feature were finally selected (**Figure 5**). The number of selected features was determined by the average AUC value evaluated by cross-validation. The prediction model constructed with the selected radiomic features showed a satisfactory performance in the training set with an AUC of 0.734 (95% CI: 0.653–0.815), a sensitivity of 0.661, and a specificity of 0.755 (**Figure 6A**). Also, the model performed well in the independent validation set with an AUC of 0.708 (95% CI: 0.589–0.827), a sensitivity of 0.714, and a specificity of 0.540 (**Figure 6B**). After combining radiomic features and clinical features (age, gender, smoking history, and max diameter of primary lung lesion), the final model achieved the highest AUC value of 0.755 (95% CI: 0.678–0.832), a sensitivity of 0.661, and a specificity of 0.755 in the training set, and an AUC of 0.736 (95% CI 0.618–0.854), a sensitivity of 0.750, and a specificity of 0.60 in the independent validation set. The nomogram combining radiomic score, max diameter of primary lung lesion, gender, age, and smoking history to predict OP is presented in **Figure 7**. Distribution of the predicted radiomic score is shown in **Figures 6C, D**. The mean ± std of predicted radiomic score in the two datasets for NP patients were 0.331 ± 0.097 and 0.310 ± 0.146, respectively, and for OP patients, they were 0.421 ± 0.125 and 0.447 ± 0.164, respectively. Moreover, the radiomic score in OP patients was significantly higher than NP patients in both datasets (*p* < 0.001). Furthermore, the final feature importance was calculated according to each feature’s average value of importance in a single tree of the GBDT model. As can be seen from **Figure 5**, the wavelet first-order statistics, wavelet GLRLM, and original GLCM texture features are relatively more important for the failure site prediction.

**Table 2:** Comparison of clinical characteristics of patients between training set and validation set.

Characteristic	Training Set (*n* = 154)	Validation Set (*n* = 79)	Statistical Method and *p*-Value
**Gender, *n* (%)**			
** Male**	58 (37.7%)	29 (36.7%)	Pearson *χ* ^2^ *p* = 0.502
** Female**	96 (62.3%)	50 (63.3%)	
**Age (years)**			
** Median Age**	61	61	Independent *t*-test *p* = 0.863
** Range**	26–84	25–79	
**Smoking History, *n* (%)**			
** Smoking**	34 (22.1%)	16 (20.3%)	Pearson *χ* ^2^ *p* = 0.866
** No Smoking**	120 (77.9%)	63 (79.7%)	
** PFS (months)**			
Median PFS	9	9	Log-rank test *p* = 0.582
Range	1–36	1–28	
**OS (months)**			
Median OS	39	42	Log-rank test *p* = 0.917
Range	1–95	6–88	
**Max Diameter of Primary Lung Lesion (cm)**			
Median Diameter	3.8	3.6	*t*-test *p* = 0.229
Range	0.9–8	0.8–10.8	
>3.25 cm	97 (63.0%)	48 (60.8%)	Log-rank test *p* = 0.740
≤3.25 cm	57 (37.0%)	31 (39.2%)	
**N Stage**			
** N0**	18 (11.7%)	7 (8.9%)	Log-rank test *p* = 0.656
** N1–3**	136 (88.3%)	72 (91.1%)	
**EGFR Mutation Site**			
Exon 19	91 (59.1%)	39 (49.4%)	Pearson *χ* ^2^ *p* = 0.308
Exon 21	58 (37.7%)	38 (48.1%)	
Uncommon Sites	5 (3.2%)	2 (2.5%)	
**First Progression Site**			
Progression in Original Lesions	56 (36.4%)	28 (35.4%)	Pearson *χ* ^2^ *p* = 1.000
Progression New Lesions	98 (63.6%)	51 (64.6%)	
**Progression Pattern**			
Oligoprogression	49 (31.8%)	27 (34.2%)	Pearson *χ* ^2^ *p* = 0.768
Systemic Progression	105 (68.2%)	52 (65.8%)	
**Metastatic sites at initial diagnosis**			
Pulmonary Metastasis	101 (65.6%)	53 (67.1%)	Pearson *χ* ^2^ *p* = 0.053
No Pulmonary Metastasis	53 (34.4%)	26 (32.9%)	
Hepatic Metastasis	13 (8.4%)	6 (7.6%)	Pearson *χ* ^2^ *p* = 1.000
No Hepatic Metastasis	141 (91.6%)	73 (92.4%)	
Osseous Metastasis	86 (55.8%)	45 (57.0%)	Pearson *χ* ^2^ *p* = 0.871
No Osseous Metastasis	68 (44.2%)	34 (43.0%)	
Cerebral Metastasis	66 (42.9%)	25 (31.6%)	Pearson *χ* ^2^ *p* = 0.119
No Cerebral Metastasis	88 (57.1%)	54 (68.4%)	
Adrenal Metastasis	13 (8.4%)	4 (5.1%)	Pearson *χ* ^2^ *p* = 0.433
No Adrenal Metastasis	141 (91.6%)	75 (94.9%)	
Plural Effusion	89 (57.8%)	38 (48.1%)	Pearson *χ* ^2^ *p* = 0.168
No Plural Effusion	65 (42.2%)	41 (51.9%)	

## 4 Discussion

Few studies have explored whether radiotherapy could improve the prognosis of patients with systemic metastasis (14). Given that the prognosis of patients with systemic metastasis was not inferior to that of patients with oligometastasis (15), it was worthwhile to explore whether radiotherapy could increase the PFS and OS for patients with systemic metastasis receiving first-line TKI treatment. Analyzing the first progression sites of first-line TKI treatment could help identify who might benefit from radiotherapy (6, 8). Our results suggested that 36.1% of patients with systemic metastasis would develop OP, who had a longer PFS and OS than those with NP after first-line TKI treatment and, therefore, should be treated differently from those developing NP. How to accurately predict the first progression sites and distinguish patients developing progression in original lesions is the prerequisite for the prescription of radiotherapy.

Radiomics is a useful technology that has been widely utilized in medical fields to excavate undiscernible biological information contained in CT images (9, 16). Radiomics has achieved good performance in the detection of biological characteristics and prediction of biological behavior of thoracic malignancies, such as distinguishment of malignant pulmonary nodules (17–19), judgment of driven gene mutational status of lung adenocarcinoma (20–22), and prediction of TKI efficacy (23). Based on this, we proposed to utilize radiomics to predict the first progression sites of first-line TKI treatment of EGFR-mutant lung adenocarcinoma patients. In our study, we finally selected seven radiomic features and four clinical features to establish a model to predict the progression sites of first-line TKI treatment. The seven selected radiomic features include transformed or original image-based texture and first-order statistical features. This indicates that the gray-scale value distribution and latent texture features inside the tumor are informative for tumor’s failure site prediction. The prediction efficacy of the established model could satisfy the basic clinical requirements to recognize patients who would develop original lesion failure and instruct the prescription of radiotherapy to them.

To the best of our knowledge, this is the first study to explore the potential candidates who might benefit from radiotherapy among EGFR-mutant lung adenocarcinoma patients with systemic metastasis. Our results demonstrated that there existed 36.1% of patients who may benefit from radiotherapy and provided a radiomic model to recognize them before treatment. However, our study still has some limitations: (a) This study has a single-institution retrospective data, which have uncertainties to extrapolate the results. (b) It has a relatively limited sample size. (c) The prediction efficacy of the established radiomic model warrants perspective multi-institutional validation. Survival improvement should be the gold standard for the validation of the established radiomic model. (d) We only utilized the radiomic features of primary lung lesions to establish the model but neglect the radiomic features of metastatic lesions. Although radiomics-based CT image analysis could provide abundant information for clinical decision-making, comprehensive analysis of CT images and biological information like genetic profiling may further improve the prediction efficacy (24). Future studies should include the radiomic features of metastatic lesions and biological information.

## Conclusion

Among patients with systemic metastasis, 36.1% developed first progression in original lesions after first-line TKI treatment and had a better prognosis than those developing first progression in new lesions. Patients with OP may be potential candidates of radiotherapy, and the radiomics prediction model might help to identify them.

## Data Availability Statement

The datasets presented in this article are not readily available because the datasets are privately owned by Shanghai Chest Hospital and are not made public. Requests to access the datasets should be directed to XF, xlfu1964@hotmail.com.

## Ethics Statement

The studies involving human participants were reviewed and approved by The First Affiliated Hospital of USTC. The ethics committee waived the requirement of written informed consent for participation.

## Author Contributions

XF, DQ, RH, and XL contributed to the study concept and design. XL and RH contributed to acquisition of data. RH, XL, XZ, WY, HL, and YY contributed to analysis and interpretation of data. XL and RH contributed to drafting of the manuscript. The corresponding author had full access to all of the data and took full responsibility for the veracity of the data and the statistical analyses. All authors contributed to the article and approved the submitted version.

## Funding

This work was supported by the Fundamental Research Funds for the Central Universities (Grant No. WK9110000177) and the Major Research Plan of the National Natural Science Foundation of China (Grant No. 92059206).

## Conflict of Interest

The authors declare that the research was conducted in the absence of any commercial or financial relationships that could be construed as a potential conflict of interest.

## Publisher’s Note

All claims expressed in this article are solely those of the authors and do not necessarily represent those of their affiliated organizations, or those of the publisher, the editors and the reviewers. Any product that may be evaluated in this article, or claim that may be made by its manufacturer, is not guaranteed or endorsed by the publisher.
